# Muopause sounder to derive vertically averaged atmospheric temperature underneath the muopause with the distance of flight muography technique

**DOI:** 10.1038/s41598-025-09090-z

**Published:** 2025-07-17

**Authors:** Hiroyuki K. M. Tanaka

**Affiliations:** 1 University of Tokyo, Tokyo, Japan; 2 International Virtual Muography Institute (VMI), Global, Tokyo, Japan

## Abstract

A number of papers have focused on monitoring surface temperature to evaluate the greenhouse effect. However, it is well known that measuring the net radiation from the entire troposphere using vertically averaged temperature would be preferable as an index to evaluate the greenhouse effect. Thus far, upper-air weather balloon systems, satellite-mounted hyperspectral infrared sounders, and microwave sounders have been used for acquiring vertically averaged tropospheric temperature. It would be advantageous to have another independent method to derive variations in vertically averaged tropospheric temperature (separate from balloon, infrared/microwave sounder measurements) to diversify the options that allow researchers to compare tropospheric temperature datasets with more variety. This paper presents the first demonstration result from a muopause sounder prototype that applies the distance of flight muography technique to measure the vertically averaged tropospheric temperature (surface = 70 hPa). The resultant relative temperature gauging accuracies of the muopause sounder prototype were respectively 1.2 K and 0.2 K for daily-averaged and monthly-averaged data. These muopause sounder’s accuracies were evaluated by comparing with measurement data acquired from a reference weather balloon station. Muopause sounders can be cheaply produced and installed into drifting buoys. The concept and design of a future large-scale muopause sounder array, suitable for enhancing spatial coverage of measurements to the global scale, is proposed.

## Introduction

Human activities, particularly the consumption of fossil fuels and the increased use of greenhouse gases, have been linked to changes in the composition of the Earth’s atmosphere and changes in global temperatures. With higher greenhouse gas concentrations in the atmosphere, thought to be mainly caused by carbon dioxide emission, “increased radiative forcing” (occurring at a rate of about 0.3 W m^-2^/decade) has been observed^1^. A number of papers have focused on monitoring surface temperature^2–8^ to evaluate the greenhouse effect; however, it is well known that measuring the net radiation from the surface of the Earth to outer space is an indirect measurement of the average temperature present throughout the troposphere, and temperatures vertically averaged below tropopause would be preferable as an index to evaluate the heat radiation processes from the Earth; hence the greenhouse effect^9,10^, and such measurements have been applied to studies on El Niño^9^ and large-scale atmospheric warming pattern monitoring in North Africa^10^.

In order to derive the vertically averaged tropospheric temperature, vertical temperature profiles of the atmosphere have traditionally been used; upper-air weather balloon systems are the most well-established method for measuring the vertical temperature profiles of the atmosphere. Each balloon carries a pressure gauge and thermometer, and the vertical profiles of pressure and temperature as well as humidity and wind direction are measured^11^. Some weather balloons are equipped with GPS receivers instead of pressure gauges^12^. Today, most of these balloons are launched using automatic balloon launchers (ABLs)^13^, but the operating costs are still high; therefore, it usually only possible to conduct sparse upper-atmosphere observations. Meteorological satellites equipped with infrared sounders^14^ and microwave sounders^15^ have operated as a possible strategy to enhance the spatial coverage: satellite-mounted sounders have the advantage of sweeping the global atmosphere above clouds (infrared), and inside and below clouds to some extent (microwave). It would be advantageous to add an independent method to the balloon and satellite measurements to diversify the data options available for researchers to compare for improvements in the reliability of tropospheric temperature measurements.

**Fig. 1:**
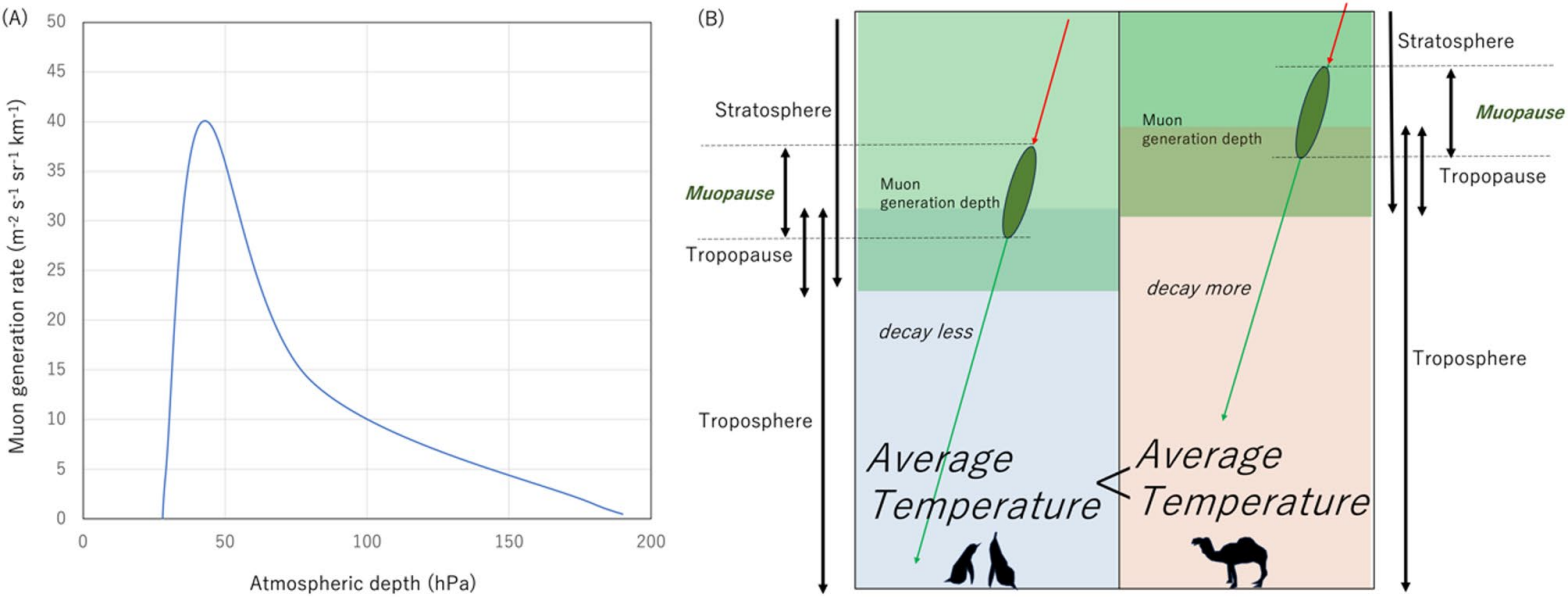
Principle of the distance of flight muography technique. The muopause is shown in a form of the muon generation rate as a function of the atmospheric depth (**A**). The data were taken from Tanaka (2025)^16^. Since the muopause height is interlinked to the geopotential height, variations in the temperature vertically averaged over the range between the detector’s height and the muopause height can be derived by measuring variations in the muon’s survival rate (**B**).

Similar to the way the Earth is divided into standard main layers such as the atmosphere, geosphere, hydrosphere, and biosphere, the muosphere is defined as the region of the Earth where abundant cosmic-ray muons (≥ 10 muons m^-2^ s^-1^ sr^-1^) are generated and exist^16^. Accordingly, the muopause is defined as the altitude region where the muons are extensively generated^16^ within an atmospheric range between 30–200 hPa (Fig. 1A). Therefore, the muopause spans the height range from the lower stratosphere to the upper troposphere. Above an atmospheric depth of 30 hPa, the muon flux is measured to be less than 0.01% of the sea level muon flux^17^. In general, the muopause height is unstable, ascending and descending depending on the atmospheric temperature located underneath the muopause.

The distance of flight (DOF) muography technique measures the ascent and descent of the muopause by observing the relative variations in muon survival rate at sea level^16^. For example, if the muopause is first upheaved and then subsequently lowered, the distance the muons travel is first extended (which has an immediate impact on decreasing the muon survival rate), and then subsequently the distance the muons travel is shortened (and the muon survival rate increases); hence, the averaged geometrical height of the muopause can be derived by gauging the muon’s survival rate as it reduces or increases (muopause height effect). The muon survival rate also depends on the average atmospheric density (atmospheric pressure) along the muon path. This effect is called the barometric effect. In the previous work, Tanaka (2025)^16^ quantitatively evaluated the muon survival rate, and found that the influence of this effect to the sea level muon flux is smaller than the muopause height effect.

As is described later, the geopotential height is proportional to the temperature averaged over the range between the ground surface and the corresponding isobaric surface. Inversely, vertically averaged tropospheric temperature can be derived from the geopotential height. This paper presents the first result of vertically averaged tropospheric temperature observations with a new and cost-effective methodology, called the “muopause sounder,” which is designed to work with the distance of flight (DOF) muography technique. This methodology is entirely independent from and complementary to (i.e., can work in tandem with) weather balloons and/or infrared/microwave sounders. A scheme for the future global deployments of the global muopause sounder array (MSA) is also proposed.

## Results

### Distance of flight muography technique

Figure 1B shows the principle of the distance of flight muography technique. Due to the muon’s time dilation by a Lorentz factor of *γ*, the muon’s decay length (*Λ*) is extended such that:

1 $$\it \Lambda = \gamma c\tau .$$

Therefore, the muon flux is reduced by an inflight decay attenuation factor (*λ*) of:

2 $$\lambda = {\text{exp }}( - \zeta /\gamma c\tau {\text{cos}}\theta ),$$

while they travel for a distance of *ζ*/cos*θ*, where *ζ* is the muon generation height (geometric), *c* is the speed of light in vacuum, *τ* is the muon’s decay constant, and *θ* is the muon’s slant angle from the zenith. Then, the attenuation factor < *λ*_0_ > averaged over various muons traveling at a slant angle of *θ* can be expressed by:

3 $$< \lambda_{0} > = {\text{ exp }}( - < \zeta > / < \gamma > c\tau {\text{cos}}\theta ),$$

where < *ζ* > and < *γ* > are respectively the averaged muon generation height and the averaged Lorentz factor of these muons. If the muopause height is varied by ± *δζ*, the averaged attenuation factor < *λ*_0_ > is modified to:

4 $$< \lambda_{1} > = {\text{ exp[}} - {(} < \zeta > \pm \delta \zeta )/ < \gamma > c\tau {\text{cos}}\theta ],$$

Consequently, the flux ratio between before and after the muopause height variation is:

5 $$N_\text{r}=N_{\mu 0} /N_{{\mu {1}}} = {\text{exp[}} \pm \delta \zeta / < \gamma > c\tau {\text{cos}}\theta ].$$

For *δζ* <<* γcτ* cos*θ*, Eq. (5) can be approximated by a linear function of *δζ*:

6 $$N_\text{r} \approx {1} \pm \delta \zeta / < \gamma > c\tau {\text{cos}}\theta ,$$

where *γcτ* cos*θ* is in the order of 10^4^ m, and *δζ* is in the order of 10^2^ m, as will be described in more detail later. Therefore, it is expected that Eq. (6) is a relatively good approximation.

### Geometric height versus geopotential height

Geopotential height (*Z*) that corresponds to a geometrical height of *ζ* is expressed as:

7 $$\begin{aligned} & Z = \frac{1}{g}\mathop \smallint \limits_{0}^{\zeta } \frac{GM}{{\left( {R + \zeta } \right)^{2} }}d\zeta \\ & \quad = - \frac{GM}{g}\left[ {\frac{1}{R + \zeta } - \frac{1}{R}} \right] , \\ \end{aligned}$$

where *R* (= 6,400 km) is the equatorial radius of the Earth, and

8 $$g=\frac{GM}{{R}^{2}},$$

is the standard acceleration of gravity. Therefore, Eq. (7) is expressed as:

9 $$\begin{aligned} & - \frac{GM}{g}\left[ {\frac{1}{R + \zeta } - \frac{1}{R}} \right] \\ & \quad = - R^{2} \left[ {\frac{1}{R + \zeta } - \frac{1}{R}} \right] \\ & \quad = - \frac{{R^{2} }}{R + \zeta } + R = \frac{R\zeta }{{R + \zeta }}. \\ \end{aligned}$$

Consequently, underneath the stratosphere, the geopotential height (*Z*) is practically the same as the geometrical height (*ζ*). For example, a geopotential height (*Z*) corresponding to a geometrical height of *ζ* = 20 km is:

10 $$Z=\frac{6400\times 20}{6400+20}=19.94 \left[\text{km}\right].$$

Therefore, the geometrical height is practically the same as the geopotential height for the muopause height region. Consequently, the following relationship holds between variations in the geometrical height *δζ* and variations in the geopotential height *δZ*:

11 $$\delta \zeta \approx \delta Z$$

### Vertically averaged temperature underneath the geopotential height

The Earth’s atmosphere mostly consists of nitrogen (78.1%) and oxygen (20.9%). Both nitrogen and oxygen can be practically treated as ideal gases at the temperatures and pressures present in the atmosphere. The fraction of H_2_O, which cannot be treated as an ideal gas, is typically 0.1–0.2%, which is practically negligible. By combining the ideal gas law:

12 $$P=\rho RT,$$

with the hydrostatic equilibrium law:

13 $$dP=\rho gdZ,$$

the following formula can be derived:

14 $$\frac{dP}{P}=\frac{-g}{RT}dZ.$$

By integrating Eq. (14) over the geopotential height range between *Z* = *Z*_0_ (detector’s height) and *Z* = *Z*_1_, the following relationship can be derived:

15 $${Z}_{1}-{Z}_{0}=\frac{RT}{g}\text{ln}\left(\frac{{P}_{0}}{{P}_{1}}\right)=\frac{RT}{g}\text{ln}{P}_\text{r}.$$

where *P*_0_ and *P*_1_ are respectively the atmospheric pressure at the detector’s location and *Z*_1_, *T* is the average virtual temperature (henceforth referred to as simply “average temperature”) between the detector’s height and *Z*_1_, and *R* is the gas constant for dry air. Thus, if *Z*_1_, *P*_0_ and *P*_1_ are given, the average temperature between sea level and *Z*_1_ can be derived.

16 $$\delta \zeta \approx \delta Z=k\delta T =\frac{R\delta T}{g}\text{ln}{P}_\text{r}.$$

Equation (16) shows that (A) there is a linear relationship between variations in the vertically averaged temperature and variations in the geometric height, and (B) the linear coefficient (*k*) varies as a function of *P*_0_/*P*_1_ (the atmospheric depth). By combining Eq. (16) and Eq. (6), we obtain:

17 $$\begin{aligned} & \delta T = \frac{gL}{R}\left( {N_\text{r} - 1} \right)\text{ln}P_\text{r}, \\ & \quad = \frac{L}{k}\left( N_\text{r} - 1 \right), \\ \end{aligned}$$

where *L *=<*γ*>*cτ*cos*θ*, and Eq. (6) is simplified to

18 $$LN_{\text{r}} \approx 1 \mp < \delta \zeta > .$$

In this work, Eq. (17) is used for reconstruction of the vertically averaged temperature from the muon data. *P*_0_ is acquired from the ground-based meteorological observations. The parameter *L* (indicating the averaged muon’s decay length multiplied by the averaged muon’s arrival angle) can be estimated by conducting EAS-MC simulations, but this method of estimation may generate a model uncertainty. Therefore, in this work, *L* was instead derived by applying the balloon measurement value of < *δζ* > and the muon measurement ratio of *N*_μ1_/*N*_μ0_ to Eq. (18). This process is called the initial calibration, and the initial part of the time sequential muon data is used for this initial calibration. After fixing the *Г* value, this value is applied to Eq. (17) (for the rest of the muon data) to derive *δζ* for the rest of the observation period.

### Measurement error

In reality, most muons are generated within the atmospheric depth range between 30 and 125 hPa. Therefore, the 1/*k* values in Eq. (16) fluctuate between 9.70 × 10^–3^ K/m (*P*_1_ = 30 hPa) and 1.63 × 10^–2^ K/m (*P*_1_ = 125 hPa) (assuming *P*_0_ = 1013 hPa). This fluctuation induces a ± 25% error of the estimated *δT.* However, by taking an average over a large number of muon events, this fluctuation is converged to the averaged value (1/*k* = 1.30 × 10^–2^ K/m at 70 hPa) according to (*K*)^1/2^, where *K* is the number of muon events to be averaged. For example, if *δT* is reconstructed by 10^4^ muon events, the estimated error level is reduced to 1/100, i.e., ± 0.25% of the estimated *δT*.

In summary, (A) the geometric height is practically the same as the geopotential height at the muopause height, and (B) the geopotential height is proportional to the vertically averaged temperature. Therefore, the temperature vertically averaged underneath the muopause can be derived with the DOF muography technique.

### Comparison between muopause measurements and ballon measurements

In this subsection, muopause sounder results are compared with the weather balloon results.

**Fig. 2:**
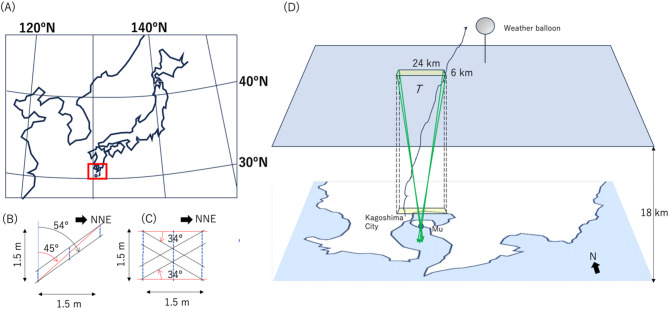
Geometric configuration of the current muopause sounder observation. The geographic location of the observation (red box) is shown (**A**). The geometric configuration of the detector is shown from the side (**B**) and from the top (**C**) with an indication of the viewing angle of the current measurements. The red solid line and the black solid lines in panel B respectively indicate the muon’s trajectories incoming at zenith angles of 45º and 54º that can be vertically accepted in the current geometrical configuration. The red solid lines and the black solid lines in panel C respectively indicate the muon’s trajectories incoming at azimuthal angles of 0º and ± 34º from NNE direction. These are the angles that can be horizontally accepted in the current geometrical configuration. The viewing areas at the height of the muopause (70 hPa) are also shown (**D**). “Mu” indicates the location of the current muopause sounder prototype. Green arrows indicate the muon trajectories with vertically and horizontally acceptable angles. Two yellow boxes indicate the current observation region at the muopause level and the ground level. The average temperature (*T*) within the cuboid formed between these two yellow boxes were measured in this work. A typical trajectory of the weather balloon is also shown.

The observation region that was chosen for the current study was Kagoshima (located in 32ºN, 131ºE) in Japan (Fig. 2A) since the weather balloon data were available in this region. In order to compare the muopause sounder results with the balloon results acquired in Kagoshima region by Japan Meteorological Agency (JMA), the muon’s arriving angles (*θ* = 45º-54º and *ϕ* =  ± 34º) were chosen in order to focus on the expected weather balloon trajectory (Fig. 2B–D). Since these balloons are not equipped with GPS, their exact trajectories are unknown, but they typically drift westward on the jet stream by a few tens of kilometers (sometimes 100 km).

The current muopause sounder prototype consisted of threefold position sensitive detectors (PSDs). Each PSD consisted of a plastic scintillator (Eljen EJ-200) strip connected to a photomultiplier tube (PMT; Hamamatsu R7724) via an acrylic light guide. The width and the length of the plastic scintillator strip were respectively 100 mm and 1500 mm; four of these strips were horizontally aligned and fifteen of these strips were vertically aligned to form a PSD with a 0.4 × 1.5 m^2^ detection area. These PSDs were vertically arranged with a spacing of 75 cm. In order to reject electromagnetic components (such as positrons/electrons), a 10-cm thick lead slab was inserted between each PSD. The muopause sounder was directed along the NNE-SSW line. The measurement period was May 1, 2023 to March 31, 2024 (11 months). It was assumed that the value of *N*_0_ didn’t vary during this period.

**Fig. 3:**
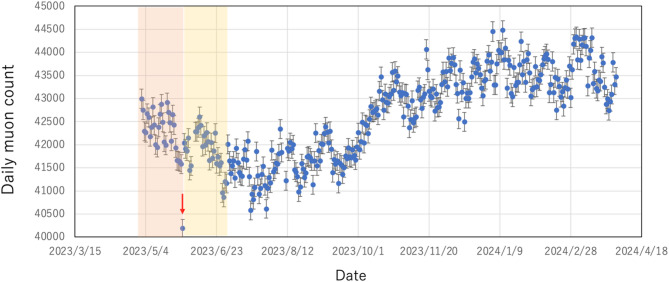
Daily muon counts collected during the current observation period (May 1, 2023–March 31, 2024). The error bars associated with the data points indicate the statistic error (1 SD). The red arrow indicates a drop in the muon count due to a power failure between 13:50 and 14:30, May 29, 2023. The pink-colored (May 1, 2023–May 31, 2023) and yellow-colored (June 1, 2023–June 30, 2023) shaded regions indicate the time regions used for the initial calibration.

Figure 3 shows the daily muon count measured during the current observation period. On May 29, 2023, there was a power failure between 13:50 and 14:30 (40 min), the data collection had been halted during this time period, and a resultant slight drop (by ~ 3%) in the muon count can be seen on the corresponding day in this plot. This data point was excluded from the current data analysis. The number of muons in each bin used for deriving the vertically averaged temperature exceeded 4 × 10^4^ events.

**Fig. 4:**
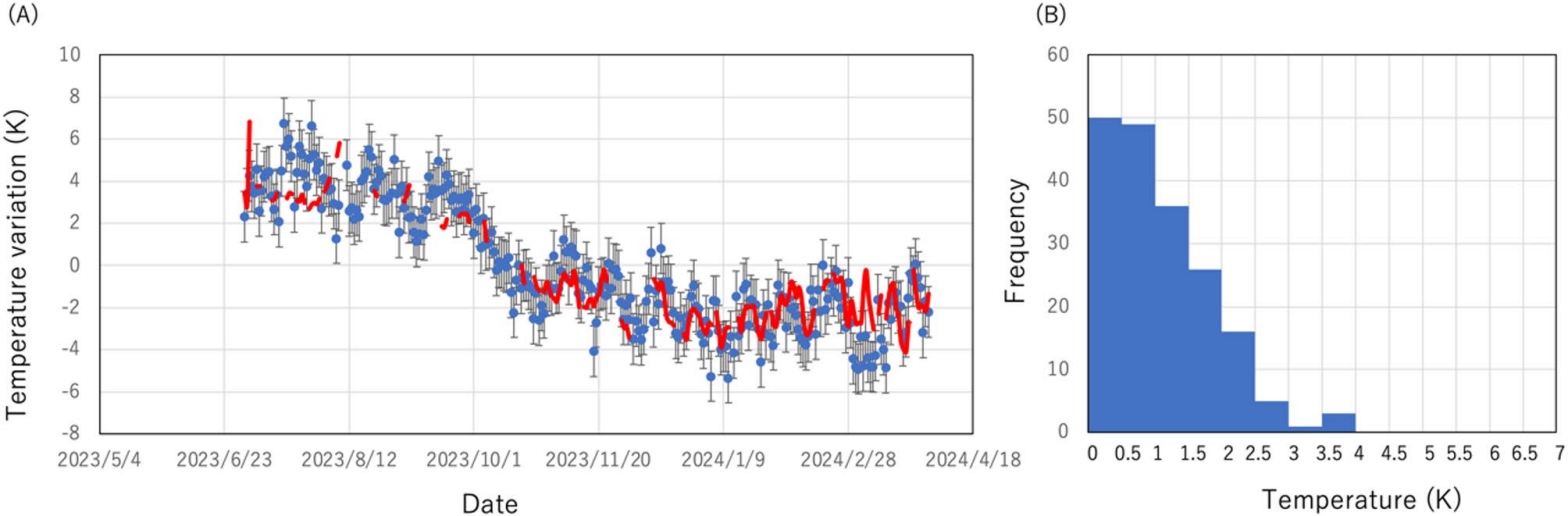
Comparison of the vertically averaged temperature between the muopause measurements and the balloon measurements. The time sequential plot of the muographically reconstructed vertically averaged temperature (small blue circles) is shown as a deviation from the average along with the balloon measurement results (red lines) within the period between July 1, 2023–March 31, 2024. The balloon measurement data were taken from Japan Meteorological Agency measurements^18^. The error bars associated with the muographic data indicate the 1*σ* statistical error. The disconnections of the red lines indicate the data missing due to an instrumental failure of the balloon operation. The absolute values of the difference (distribution) of the vertically averaged temperature between the balloon-based measurements and muographic measurements are also shown (**B**).

Figure 4A compares seasonal variations in the (daily) vertically averaged temperature which were derived by applying Eq. (17) to the muon data shown in Fig. 3 and seasonal variations in the vertically averaged temperature derived from the JMA balloon-based measurement data^18^. This plot was produced based on the following procedure:

(A) The daily averaged muon flux data were first barometrically corrected according to the procedure reported in Tanaka (2025)^16^ by using the following relationship: *ΔN* = *kΔP* + *C*, where *ΔN* and *ΔP* are respectively variations in the muon flux and the atmospheric pressure, and the constants *k* and *C* are respectively − 1.1955 × 10^–3^ and 2.2159.
(B) The *Г* value of Eq. (18) was derived by inserting < *N*_μ1_*>*, < *N*_μ0_*>*, and < *δζ*> as obtained from the weather balloon measurements. In this work, the average of *N*_μ1_ was taken over the period between May 1, 2023 and May 31, 2023, the average of *N*_μ0_ was taken over the period between June 1, 2023 and June 30, 2023, < *δζ*> was computed by subtracting the geopotential height at 70 hPa averaged over the time period between May 1, 2023 and May 31, 2023 from the geopotential height at 70 hPa averaged over the time range between June 1, 2023 and June 30, 2023. Ground surface averaged atmospheric pressures were respectively 1,009 hPa and 1,005 hPa in May, 2023 and June, 2023.
(C) After this initial calibration, the derived *L* value was inserted into Eq. (17), and the daily averaged *δT* values were derived for the rest of the observation period (July 1, 2023–March 31, 2024) as a deviation from the *δT* value averaged over this time range. The daily averaged *P*_0_ data were acquired from Kagoshima meteorological station^18^. *P*_1_ was fixed to be 70 hPa.

70 hPa (18 km a.s.l.) is representative of the pressure level of the muopause in terms of the atmospheric depth and the muon survival rate. The muon survival rate varies between 125 and 30 hPa within the muopause. The averaged muon survival rate between 15 km a.s.l. (125 hPa) and 24 km a.s.l. (30 hPa) is:

19 $$\begin{aligned} & \left[ {{\text{exp}}\left( { - \frac{{15 \left[ {{\text{vertical }}\;{\text{height }}\;{\text{at }}\;125\;{\text{ hPa}};{ }\;{\text{km}}} \right]}}{{6.6{ }\left[ {{\text{muon }}\;{\text{decay }}\;{\text{length}};\;{\text{ km}}} \right]}}} \right)} \right. \\ & \quad + {\text{exp}}\left. { - \frac{{24 \left[ {{\text{vertical }}\;{\text{height }}\;{\text{at}}\;{ }30{ }\;{\text{hPa}};\;{\text{ km}}} \right]}}{{6.6{ }\left[ {{\text{muon}}\;{\text{ decay }}\;{\text{length}};{ }\;{\text{km}}} \right]}}} \right]/2 = 0.065 \\ \end{aligned}$$

and the muon survival rate at the representative muopause height of 18 km a.s.l. (70 hPa) is:

20 $$\text{exp}\left(-\frac{18 \left[\text{vertical height at }70\text{ hPa};\text{ km}\right]}{6.6 \left[\text{muon decay length};\text{ km}\right]}\right)=0.065$$

Here, the typical cosmic-ray muon decay length for *γ*  = 10 at 70 hPa^17^ was employed as the muon decay length in Eqs. (19) and (20).

The averaged muon events in June, 2023 and in May, 2023 were respectively 41,838 ± 37 (1 SD) events per day and 42,246 ± 38 (1 SD) events per day, thus the ratio, < *N*_μ1_*>*/< *N*_μ0_*>*, was 0.990362. On the other hand, the averaged geopotential height (< *ζ* >) at 70 hPa in May, 2023 and June, 2023 were respectively 18,653 m and 18,752 m. Therefore, their difference (< *δζ* >) was 99 m. Consequently, the derived *L* value was 10,344 m. Considering the averaged *θ* of ~ 50º, the averaged muon’s decay length at 70 hPa is ~ 7 km (averaged *γ*  ~ 10) which is consistent with the averaged muon energy (~ 1 GeV) at 70 hPa measured in the prior work^17^.

In Fig. 4B, the difference (distribution) between the balloon-based measurements and muographic measurements is shown. The muographic results are basically consistent with the balloon results, with a standard deviation of 1.3 K which is close to a 1*σ* statistic error (~ 1.2 K) associated with the muographic data points. This standard deviation is reduced to ~ 0.8 K (1*σ* statistic error: ~ 0.4 K), ~ 0.6 K (1*σ* statistic error: ~ 0.3 K), and ~ 0.4 K (1*σ* statistic error: ~ 0.2 K) respectively by taking an average over 10 days, 20 days, and 30 days.

**Fig. 5:**
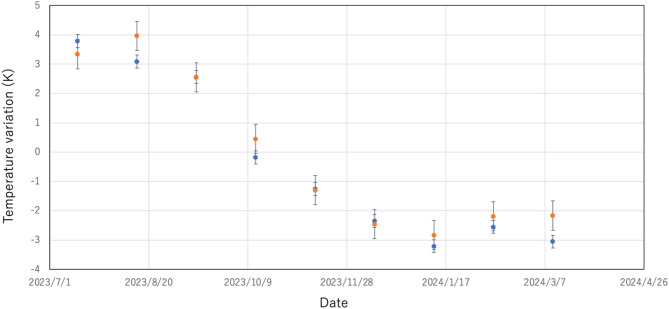
Comparison between the monthly averaged muographic results (blue circle) and the monthly averaged balloon results (orange circle). The error bars associated with the muographic data points indicate statistic errors. Although the errors associated with balloon results have not been made public by JMA, the tentative error (0.5 K) based on the report by WMO^19^ is indicated by the error bars associated with the balloon data points as a typical value.

In Fig. 5, the monthly averaged muographic results are compared with the monthly averaged balloon results. 7 data points out of 9 data points are in agreement within 1*σ* error bars, and the other 2 data points are in agreement within 2*σ*. Consequently, these muographic results are statistically in agreement with the balloon results (in accordance with the normal distribution probability density function (PDF)).

As shown in Fig. 2, muon generation points are distributed in horizontally uniform within the muopause and thus, a muopause sounder measures the temperature averaged over a given cuboid region. On the other hand, a weather balloon measures the temperature averaged along its each trajectory which often varies from day to day. This is the essential difference between the muopause sounder and weather balloon and thus, the muopause sounder is well suited to the current purpose (to measure the average bulk temperature of the atmosphere.) The current comparison between the daily muopause sounder data and the daily weather balloon data is based on an assumption that average tropospheric temperature doesn’t vary drastically within a local region and within a special scale of tens of kilometers. This assumption is consistent with the fact that the weather balloons generally used for regular observations are not equipped with GPS antennas. Although weather balloons can sometimes drift away for tens of kilometers, the fact that the measurements taken by the weather balloon are representative of the values ​​at the launching position is likely due to the same assumption. As is described in the next section, a reasonable statistical agreement between the deviation of the daily muopause sounder data from the daily weather ballon data and the deviation of the monthly averaged muopause sounder data from the monthly averaged weather ballon data further indicates that this assumption is reasonable (since weather balloon trajectories are monthly averaged to indicate closer values to the average bulk temperature).

## Discussion

The standard deviation (SD) between the muographic measurement results and the balloon measurement results is 1.3 K (1 SD) which is close to the statistic error (~ 1.2 K) associated with the muographic data, but as previously shown, this SD is not reduced according to *K*^-1/2^, where *K* is the number of data points to be averaged. This is probably due to a balloon measurement error. WMO reported that most modern radiosonde systems measure temperature in the troposphere with a standard error between 0.2 and 0.5 K^19^. The aforementioned SD reduction pattern as a function of *K* can be explained if the balloon measurement error is assumed to be ~ 0.5 K.

In order to reduce this statistic error (1.2 K) of daily muographic measurements enough to match the weather balloon error measurement (0.5 K) level, either a reduction of the time resolution by a factor of 6 or an enlargement of the detection area by a factor of 6 will be needed. The latter option is probably impractical if applied to a large-scale muon detector array in the global muopause sounder array (MSA) which will be proposed in the next subsection. If the purpose of global MSA is to observe long term trends of the climate change, a monthly time resolution would be sufficient^9^.

### Ocean-based muopause sounder network

**Fig. 6:**
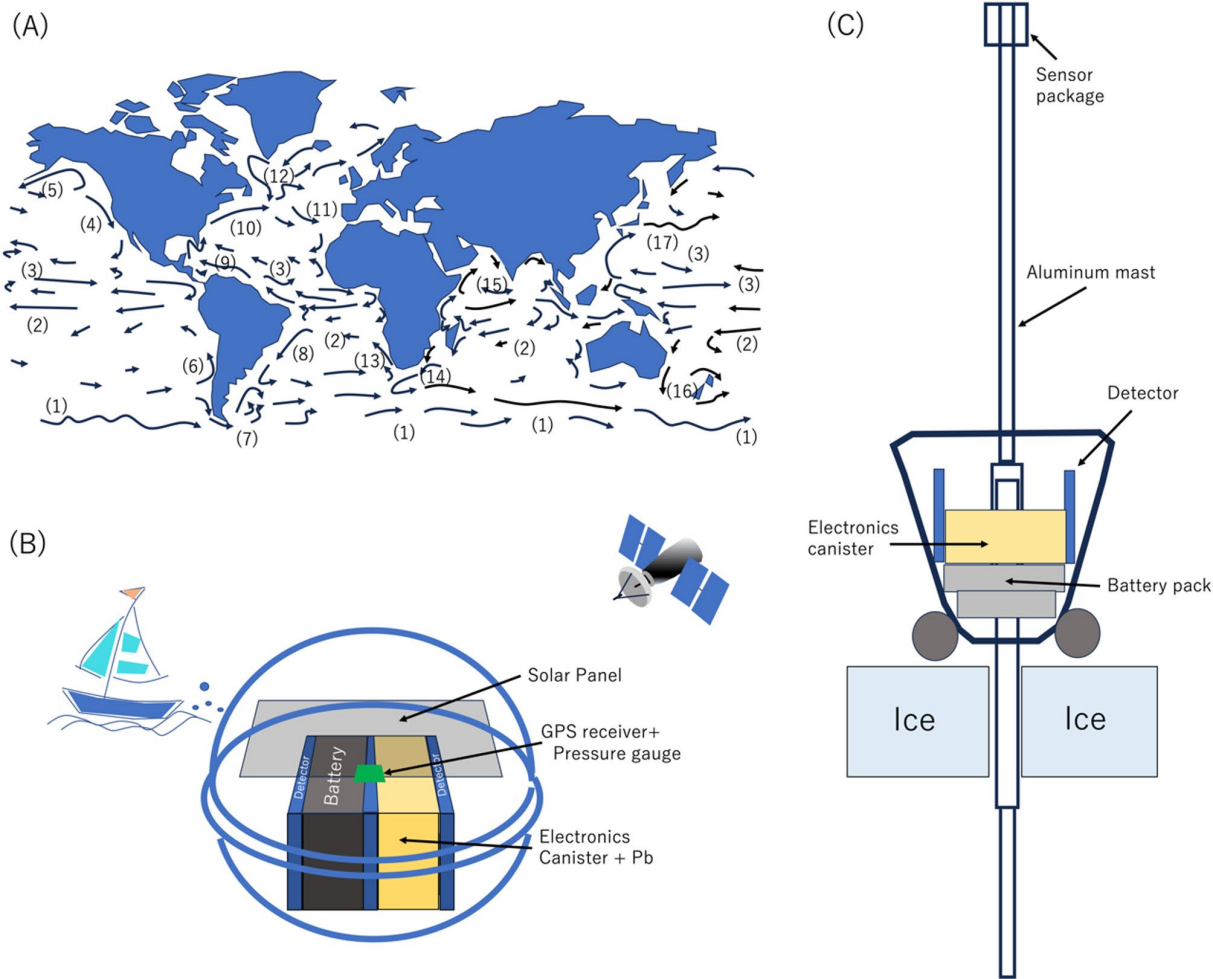
Scheme of the global MSA setup. The surface currents are shown (**A**). The numbers in panel A indicate the names of the currents: 1) Arctic Circumpolar, 2) South Equatorial, 3) North Equatorial, 4) Californian, 5) Alaskan, 6) Peru/Chile, 7) Malvinas, 8) Brazil, 9) Caribbean, 10) Gulf Stream, 11) Azores, 12) North Atlantic, 13) Benguela, 14) Agulhas, 15) Monsoon, 16) East Austral, and 17) Kuroshio^23^. The proposed ocean-based muopause sounder equipped with a drifting buoy is also shown (**B**). A possible scheme to install a muopause sounder into Airborne Expendable Ice Buoys is also shown (**C**).

Currently, only satellite-based methods are now capable of making vertically averaged temperature observations for regions above the ocean. It might be advantageous to deploy global MSA (by installing them into drifting buoys) to offer complementary global temperature data to the satellite data. The basic concept is that the global MSA units would drift on the surface current (Fig. 6A). The detection area required to attain an error level of 0.5 K in one month would be 1,200 cm^2^ which is smaller than the largest cross-sectional area of the drifter surface float (1,256 cm^2^) used in the National Oceanic and Atmospheric Administration (NOAA)’s Global Drifter Program (GDP)^20^.

A schematic drawing of the possible setup for this type of measurement is shown in Fig. 6B. The power consumption of each muopause sounder is less than 1 W (SiPM; 150 mW × 3, comparators and coincidence circuits are very small) and thus, it can be operated for more than 6 months (even if solar power is not available) using only a 5-kWh lithium battery (volume, 25,000 cm^3^, weight 37 kg). As a relevant model for this approach, here we briefly introduce the drifter array used in the NOAA’s GDP. GDP maintains a global 5° × 5° gridded array of ~ 1,300 satellite-tracked surface drifting buoys that measure air pressure over the ocean and send these data to satellites passing overhead, which then transmit the data within one hour to weather forecasters^21^. The diameter of the surface float ranges from 30.5 cm to 40 cm, and weighs ~ 25 kg^20^. Their standard Surface Velocity Program (SVP) drifter costs roughly 1,800 USD^20^. Since the horizontal position of the floats could be monitored with satellite positioning systems, the exact positions of each drifter would be possible to fix precisely. There are enough similarities between the proposed global MSA and these global drifters to benefit from investigating the experience of the GDP team.

Standard drifter devices can be used in most ocean environments, however for the environment of the Arctic Ocean, a more complex setup would be required. In the Arctic Ocean, muopause sounders can be installed within an Airborne Expendable Ice Buoy (AXIB)^22^. The AXIB can be dropped from an airborne platform, and is designed to land on an ice surface, align itself to a vertical position, and then anchor and stabilize itself in the ice. Since it has a 53 cm diameter and 94 cm height, a 1,200 cm^2^ tracker can be accommodated on the AXIB-type float. The possible scheme of a muopause sounder installation within the AXIB-type framework is shown in Fig. 6C.

### Costs

The costs required to build the muopause sounder prototype used in the current demonstration are summarized as follows: (A) wrapped plastic scintillator strips, 300 USD × 53 = 15.9 k USD, (B) photomultiplier tubes, 500 USD × 53 = 26.5 k USD, (C) lead blocks, 4.3 k/ton × 0.88 tons = 3.8 k USD, (D) electronics and cables, 10 k USD, and (E) a frame, 20 k USD. Therefore, the total cost is 76.2 k USD. This setup might be suitable for ground-based measurements (less than 1/4 of the costs for the annual operation of the balloon measurements (Table 1) even if the actual annual muopause sounder operation costs are included) to cover the area where the balloon measurements cannot be deployed or to act as a backup system, but it is not an optimal or ideal solution if designing an MSA for global measurements.

**Table 1:** Performance comparison.

	Accuracy (K)	Time resolution (day)	Spatial resolution (km)	Cost (USD)	Observable quantities
Weather Balloon	0.2–0.5^19^	Launching frequency (~ 1)	Drifting distance at horizontal (~ 100 at the maximum), continuous vertical	380 k/year (operation)^26^	Temperature, humidity, wind profiles
Infrared sounders					
AIRS	0.1–0.6^27^	Real time (16*)^28^	13.5 at horizontal at nadir, 1 vertical^29^	220 M^+^ (deployment)^30^ 3 M/yr^+++^ (operation)^31^	Temperature, and humidity profiles
Microwave sounders					
SAPHIR	0.5–1.4^32^	Real time (7**)^33^	10 at horizontal at nadir^34^	120 M^++^ (deployment)^35^ 3 M/yr^+++^ (operation)^31^	Temperature, and humidity profiles
ATMS	0.8–0.9^32^	Real time (16***)^36^	16–75 at horizontal at nadir^37^	120 M^++^ (deployment)^35^ 3 M/yr^+++^ (operation)^31^	Temperature, and humidity profiles
MHS	0.7–1.1^32^	Real time (11****)^38^	15 at horizontal at nadir^39^	120 M^++^ (deployment)^35^ 3 M/yr^+++^ (operation)^31^	Temperature, and humidity profiles
Muopause Sounders					
Prototype (This work)	0.2–1.2	1–30	6–24 at horizontal, averaged underneath the muopause vertical	76 k (deployment) 20 k/yr^++++^ (operation)	Vertically averaged temperature underneath the muopause
Global MSA (proposed)	0.5	30	~ 500^#^ (5º × 5º)	2.8 M^+++++^ (deployment) 20 k/yr^++++^ (operation)	Vertically averaged temperature underneath the muopause and neutron flux

*Repeat cycle of Aqua satellite.

**Repeat cycle of Megha-Tropiques satellite.

***Repeat cycle of Suomi-NPP satellite.

****Repeat cycle of NOAA-18 satellite.

^#^Assuming the same configuration of GDP.

^+^Typical deployment costs for an infrared sounder satellite.

^++^Typical deployment costs for a microwave sounder satellite.

^+++^Typical costs for a meteorological satellite annual operation.

^++++^Actual costs outsourced to an external IT company for annual operation of the current muopause sounder prototype used in this work including the server rental costs, maintenance costs, electric and internet costs.

^+++++^Assuming 1,000 OMS deployments.

The costs required to produce three layers of 1,200 cm^2^ single channel detectors are less than 1 k dollars (SiPM: 20 USD × 3 + plastic scintillator sheet: 200 USD × 3 + associated electronics 200 USD). Therefore, by assuming the use of a standard SVP-type drifter (which would operate well in almost all ocean environments) as a platform of the muopause sounder, the total cost for each drifter system could be reduced to less than 2,800 USD. This particular kind of cost-effective drifter system could be easily replicated, and would make it possible for researchers to distribute more widely to enhance spatial coverage of measurements to the global scale.

### Synergy effect with neutron monitoring

There have been debates about possible links between cosmic rays and global warming^24^. There are some rather exotic theories that indicate global warming is more affected by galactic cosmic-ray flux than by rising concentrations of greenhouse gasses^25^. They attempt to prove that there is a connection between the variations in the cosmic ray flux and variations in cloud formation and density. If the total incoming energy from the Sun is assumed to be 100%, the solar energy blocked by clouds is larger than re-radiation of the solar energy by clouds to the ground surface by 10%: (15% re-radiation to the ground) − (5% absorption into the cloud) − (20% reflection to outwards). If the amounts of clouds are increased, this excess will increase; hence the ground surface temperature decreases.

Neutron-monitor-equipped global MSA has the unique potential to distinguish whether (A) cosmic-ray changes the net cloud density then, changes the global surface temperature, or (B) the global temperature variations are due to solar activity variations, and cosmic-ray flux variations are merely a result of these solar activity variations. If (A), the tropospheric temperature is kept; hence the muopause height doesn’t change. If (B), the tropospheric temperature changes and thus, the muopause height changes. The in-situ monitoring of the cosmic-ray intensity and the local tropospheric average temperature will enable researchers to monitor the long-term trend of the latitudinal and longitudinal dependence (rigidity cutoff dependence) of the relationship between tropospheric temperature and primary cosmic-ray flux.

### Summary comparison

Table 1 shows the performance comparison between weather balloon measurements, infrared sounder measurements, microwave sounder measurements, and muopause sounder measurements (current work and the proposed global MSA). In conclusion, a temperature gauging accuracy (vertically averaged) and time resolution, which falls within the same basic range as other major techniques, was acquired. Although the time resolutions of infrared sounders and microwave sounders function at higher rates (almost real time) compared to the weather balloon and the muopause sounder, these rates decrease when focused on the same region; since the satellite is always moving, there is a certain period in which the region cannot be measured prior to the moment that the satellite returns to the same point on the Earth (repeat cycle).

### Limitations of the current work

Validations of the current concept have been conducted in Japan. Although it is expected that the proposed theory is globally applicable, additional validations will be required for regions with extremely high H_2_O concentrations, such as in very humid tropical regions. Validations in other climate zones will be the next step of this research for systematic evaluation of the muopause sounder.

The current daily muopause sounder error level is 1.2 K, which is higher than the weather balloon measurement error level (0.5 K) while the monthly-averaged muopause sounder error level (0.2 K) is lower. Such an accuracy degradation can be improved by increasing the size of the detection area of the sounder. For example, a daily time resolution at an error level of 0.2 K is achievable by expanding the size of the detection area by a factor of 30 (~ 18 m^2^) which would cost ~ 320 k USD (since the number of channels; hence building costs are roughly proportional to the square root of the detection area).

## Conclusion

In conclusion, the parameter (*Г*) could be successfully determined by using 2 months of muon data and which reproduced the vertically averaged temperature for the subsequent 9 months of muon data. As a result, favorable relative temperature gauging accuracies were confirmed with the current muopause sounder prototype. Since muopause sounding data can be collected independently from balloon-based data or satellite-based data, muopause sounding data can diversify the options available to researchers and can allow for comparisons to improve reliability of the currently available tropospheric temperature data. The muopause sounding data can also be used as a backup of the data which are obtained from these established techniques.

## Data Availability

The datasets used and/or analyzed during the current study are available from the corresponding author on reasonable request.
